# Exploring healthcare professionals’ experiences and perspectives on cessation support for people who use non-cigarette tobacco: a qualitative study

**DOI:** 10.1136/bmjph-2025-004768

**Published:** 2026-08-04

**Authors:** Eve Taylor, Harry Tattan-Birch, Jamie Brown, Lion Shahab, Sharon Cox, Masuma Pervin Mishu, Katherine East, Hazel Cheeseman, Sarah Jackson

**Affiliations:** 1 Department of Behavioural Science and Health, University College London, London, UK; 2 National Addiction Centre, Institute of Psychiatry, Psychology & Neuroscience, King’s College London, London, UK; 3 Department of Primary Care and Public Health, Brighton and Sussex Medical School, Brighton, UK; 4 Action on Smoking and Health, London, UK

**Keywords:** Primary Prevention, Health Services Accessibility, Health Personnel

## Abstract

**Introduction:**

Healthcare services in England often support cigarette smoking cessation, yet little is known about how healthcare professionals support people to quit who use other non-cigarette tobacco products, such as cigars, cigarillos, shisha and different forms of chewing tobacco. This study explored healthcare professionals’ (1) experiences of providing cessation support for non-cigarette tobacco, (2) perceived barriers and facilitators and (3) suggestions for improving service provision.

**Methods:**

Four semistructured focus groups and interviews were conducted with (N=19) specialist (tobacco dependence advisors) and non-specialist (eg, pharmacist, nurse) healthcare professionals who have experience in providing smoking cessation support across England. Data were analysed using iterative categorisation with thematic analysis.

**Results:**

Healthcare professionals vary in their experiences supporting people to stop using non-cigarette tobacco products, usually depending on the demographic composition of the local community. Professionals who regularly engage with people who use non-cigarette tobacco described a range of tailored support provided, such as behavioural support, nicotine replacement therapy and vapes. Structural barriers to providing support included screening tools that only ask about ‘smoking’, commissioning that restricts services to cigarette use, and limited training and resources for non-cigarette products. Professional barriers included low confidence in supporting cessation due to a lack of official guidance on these products. Recommendations to improve services included adapting screening tools to capture non-cigarette tobacco use, expanding service eligibility to all tobacco users, developing culturally tailored resources and strengthening community partnerships to improve outreach.

**Conclusions:**

In a sample of healthcare professionals in England, there was substantial variation in the provision of, and healthcare professionals’ confidence in providing, support for non-cigarette tobacco cessation. Amending screening tools, developing clinical training and guidelines and expanding access to services for all types of tobacco use could improve support provision and improve treatment equity.

**WHAT IS ALREADY KNOWN ON THIS TOPIC**

- A range of non-cigarette tobacco products are currently used in England, including cigars, cigarillos, pipes, bidis, shisha and chewing tobacco. Comprehensive cigarette smoking cessation support is available in England; however, little is known about the support available, or specific barriers that healthcare professionals face when supporting people to quit non-cigarette tobacco.

**WHAT THIS STUDY ADDS**

- The frequency with which specialist and non-specialist healthcare professionals encountered non-cigarette tobacco use and their knowledge of these products varied. Specialists often supported cessation with a range of nicotine replacement therapies and behavioural counselling. Key barriers in providing support included identifying use, services not being commissioned to support non-cigarette tobacco and a lack of non-cigarette-specific training and guidance.

**HOW THIS STUDY MIGHT AFFECT RESEARCH, PRACTICE OR POLICY**

- Screening in healthcare settings should be adapted to ask people about specific types of tobacco rather than just whether they smoke, and specific guidance and treatment protocols should be developed for healthcare professionals to consult when they meet people who use non-cigarette tobacco.

## Introduction

 Tobacco use is a leading cause of preventable premature death, disease and health inequalities in England.^1^ While cigarette smoking remains the most common form of tobacco use, with 12.8% of adults in England currently smoking,^2^ there are a range of other non-cigarette tobacco products used, such as cigars, cigarillos, pipes, bidis, waterpipe tobacco (shisha, hookah or narghile) and chewing tobacco (eg, paan with tobacco, gul, naswar). Approximately 3.7% of adults in England use non-cigarette tobacco products, with cigars (1.2%) and shisha (0.9%) most commonly reported.^3^ Although population-level prevalence remains low, use of certain products, such as shisha and chewing tobacco, is substantially greater among British South Asian and British Arab communities.^4^ While all tobacco use carries significant health risks compared with non-use, the level of associated harm varies across products.^5–9^ Depending on how it is made, chewing tobacco can contain fewer toxicants than cigarettes,^6,10^ while shisha and cigars are associated with similar disease risk as cigarette smoking, after accounting for differences in use patterns.^5,11,12^

It has been advised that primary healthcare professionals in England, such as general practitioners (GPs) and dentists, should assess patients’ use of non-cigarette tobacco products as well as cigarette smoking.^13^ Healthcare professionals play a key role in identifying tobacco users and referring them to specialist stop smoking services, where they can receive more intensive and tailored support. These clinical interactions also provide valuable ‘teachable moments’ for delivering cessation advice. However, such opportunities are often missed due to a range of barriers.^14^ Little is currently known about healthcare professionals’ knowledge of non-cigarette tobacco products or their referral practices. Understanding how they perceive these products, and their experiences of helping people to quit, can help inform the provision of effective support for non-cigarette tobacco cessation.

England offers comprehensive evidence-based smoking cessation support, including behavioural interventions, prescription medications, nicotine replacement therapy (NRT) and e-cigarettes (vapes).^15,16^ Support is often provided by specialist smoking cessation services, which are commissioned regionally by local authorities. Evidence for cigarette smoking cessation shows that success rates tend to be higher when people receive support from specialist stop smoking services.^17^ These services—funded by local authorities—can be delivered through dedicated centres or embedded into other healthcare settings such as pharmacies or GP practices. While some services offer support for non-cigarette tobacco, the extent of such provision remains unclear.

International evidence suggests that specialist services can support cessation among non-cigarette tobacco users. For example, face-to-face behavioural support has been shown to help people to quit shisha tobacco.^18^ A recent systematic review also found that behavioural support, pharmaceutical support (varenicline) and NRT improve cessation outcomes among people using smokeless tobacco (ie, oral or nasal tobacco that does not involve combustion or heating).^19^ UK-based interventions have likewise demonstrated that 12-week behavioural support programmes, with or without NRT, can help people quit chewing tobacco.^20^ Despite this evidence, little is known about the experiences of smoking cessation advisors and other healthcare practitioners in delivering such interventions.

Healthcare professionals may face specific barriers when supporting people to quit non-cigarette tobacco. These include limited awareness of associated health harms,^21–24^ and difficulty identifying users, particularly those who do not perceive themselves as ‘smokers’ or tobacco users, such as people who use shisha or paan.^15^ Paan, a preparation of areca nut, slaked lime and betel leaves, may or may not contain added tobacco, yet users are not always aware of whether their product includes it.^25^ Healthcare professionals may also face uncertainties about how best to engage specific groups, such as young adults who use cigars or cigarillos,^26^ or ethnic minority communities where shisha and chewing tobacco use is more prevalent.^4^ Professionals working with these populations should therefore be equipped with the knowledge, cultural competence and confidence to raise the issue of harms from non-cigarette tobacco and the benefits of quitting. However, barriers such as language differences, cultural sensitivities and mistrust of health services may hinder effective delivery of cessation support.^21^

This study explored healthcare professionals’ knowledge, experiences and perceived barriers and facilitators related to non-cigarette tobacco cessation. Specifically, we sought to answer the following research questions (RQs):

RQ1. What are the experiences of healthcare professionals and stop smoking service providers in providing cessation support for non-cigarette tobacco?
RQ2. What do healthcare professionals think influence the availability and provision of cessation services for people who use non-cigarette tobacco?
RQ3. What do healthcare professionals think could improve the availability and provision of cessation services for people who use non-cigarette tobacco?

## Materials and methods

### Participants

Participants were recruited through the National Centre for Smoking Cessation and Training (NCSCT), online adverts, snowball sampling and targeted emails to smoking cessation services. Eligible participants were required to be aged 18+, self-report that they were currently employed as a healthcare professional in England, and have experience providing smoking cessation support. All participants were asked if they had met clients who used any of the following products during screening: cigars, cigarillos, pipes, bidis, waterpipe tobacco (shisha, hookah or narghile) and chewing tobacco. Those who indicated encountering clients who used non-cigarette tobacco were prioritised for focus groups as they were deemed to be able to provide greater insight. Initially, three focus groups were planned, comprising four to six participants who were (1) tobacco dependence advisors (TDAs) from services with dedicated non-cigarette tobacco cessation services, (2) TDAs from services without dedicated non-tobacco cessation services and (3) healthcare professionals (eg, GPs, dental professionals and pharmacists) who provide stop smoking support, but it is not their main role. Due to two dropouts for each of the focus groups among TDAs, and to achieve data saturation among the non-specialist healthcare professions, two additional focus groups and three one-on-one interviews were conducted.

### Design and procedures

Semistructured focus groups and interviews were conducted by ET online between May and August 2025, lasting for between 40 and 90 min. Focus groups and interviews followed the same semistructured topic guide, which was published on the Open Science Framework.^27^ The topic guide covered (1) knowledge of non-cigarette tobacco products, (2) experiences and opinions of helping people quit, (3) barriers to providing cessation support, (4) facilitators to providing cessation support and (5) ideas on how to improve support. Video and audio recordings were captured and later transcribed by an independent third party. Participants were interviewed once and were not asked for comments on the transcription. Data saturation was determined by ET when no new topics arose from interviews. Participants were reimbursed with a £30 shopping voucher.

### Analysis

Iterative categorisation,^28^ a systematic and staged approach to qualitative data management and analysis, was used alongside thematic analysis. First, data were coded by ET in NVivo following deductive codes derived from the topic guide and agreed with SJ. Additional inductive themes and subthemes that arose from the data were also coded. Findings were organised in accordance with the research questions.

### Reflexivity

Interviews and focus groups and analysis were conducted by ET, a Research Fellow with a PhD in Addiction Science. Before conducting interviews, ET attended training on interview technique and iterative categorisation analysis. ET had no prior relationship with any of the participants, and she introduced them to the aims of the research before the focus groups and interviews were conducted.

### Patient and public involvement

Patients and the public were not consulted in the development of this research.

## Results

### Participant characteristics

Participants were from a range of different types of healthcare services, including inpatient TDAs, specialist TDAs from stop smoking services, a nutritionist, a pharmacist, nurses and a GP (table 1). Participants were recruited from regions across England, including Tyne and Wear, Staffordshire, Leicestershire and Manchester. Services in London were overly represented (approximately half of the participants).

**Table 1:** Participant characteristics

Focus group	Participants	Healthcare professions
Non-specialist healthcare professionals*		
Focus group 1	5	Nurse × 3 Nutritionist × 1 Community health and behaviour support worker × 1
Focus group 2	5	Nurse × 1 Health improvement practitioner × 1 Maternity nurse and TDA × 1 Pharmacist × 1 Dental hygienist × 1
Interview 1	1	GP with an interest in chewing tobacco
Specialist healthcare professionals†		
Focus group 3	3	TDA leads in inpatient mental health service × 2 TDA in a stop smoking service × 1
Interview 2	1	TDA in a stop smoking service
Specialist healthcare professionals with dedicated non-cigarette services†		
Focus group 4	3	TDA in a stop smoking service with non-cigarette tobacco specialism × 1 TDA lead in a stop smoking service with non-cigarette tobacco specialism × 1 TDA in a stop smoking service × 1
Interview 3	1	TDA in a stop smoking service with non-cigarette tobacco specialism

* Non-specialist healthcare professionals are those who provide support for tobacco cessation; however, it is not their main role.

† Specialist healthcare professionals are those whose main role is providing tobacco cessation support.

GP, general practitioner; TDA, tobacco dependence advisor.

### Healthcare professionals' experiences of providing non-cigarette tobacco treatment

Participants’ experience with different types of non-cigarette tobacco products varied considerably. Some were very knowledgeable about a range of products, whereas others had limited awareness and only learnt about certain products through patient interactions.

A lot of … the things we’ve learnt are through experience of them [with] patients… we are then able to come up with our own little plan of how we think we would be able to help support that patient (Focus group 3, TDA)

#### Encountering non-cigarette tobacco use

The frequency with which specialist and non-specialist healthcare professionals encountered non-cigarette tobacco use varied widely—from never meeting anyone who uses cigars, shisha or chewing tobacco to meeting people who use these products at least weekly. Participants rarely discussed meeting clients who used cigars or cigarillos; however, one participant did note that encountering people using cigars is occurring ‘far more often’.

Cigarettes, yes. Not so much cigars, no, I don’t really see that (Interview 3, TDA)

Clients who used shisha often also smoked cigarettes. Two participants who worked with pregnant people noted that they regularly (at least monthly) encountered pregnant clients using shisha.

Most of the shisha consumers that come to our service, they also smoke… it is very hard to find someone who is exclusively using non-smoked tobacco (FG4, TDA)

The prevalence of chewing tobacco use was said to depend heavily on the community being served and was described as more common among older South Asian people than other communities. For example, one specialist reported receiving ‘clients every day who would use smokeless tobacco’ when working in the West Midlands, but far fewer since moving to an area with a predominantly White British population. Unlike cigars and shisha, participants noted that women who use chewing tobacco also rarely smoke cigarettes.

Then we had a few clients who chew tobacco because we have a huge Bengali community here, so, I think that kind of practice is more isolated within community clusters (FG4, TDA)

Paan is socially acceptable. Because it’s done a lot in- back home in Bangladesh. But definitely not smoking. It’s not as common to see a woman smoking (I3, TDA)

#### Engaging with patients

Although specialist services commonly received referrals from other healthcare settings and charities, participants noted that people using non-cigarette tobacco products typically accessed support through community outreach or self-referral. For chewing tobacco in particular, two specialists reported that younger family members seeking help for smoking or vaping cessation often asked whether older relatives who used chewing tobacco could also receive support.

Mostly they are like family members of people who have come for stop smoking, and then they ask questions like, “Do you help with other products?” (FG4, TDA)

#### Identifying use

Two specialists said they routinely ask everyone about every type of tobacco that they use.

We have to ask. We have our own special form, and it’s one of the questions. What type of tobacco do you smoke? And do you have smokeless tobacco? (I3, TDA)

In contrast, a dentist and a GP explained that they only raise the topic when it is directly relevant to the appointment—for example, if a patient presents with a ‘seemingly relevant’ condition or ‘oral staining’.

One participant noted that they explicitly ask about chewing tobacco when the patient is from a certain ethnic background, and about shisha when the patient is younger. Other participants said that non-cigarette tobacco use is more often uncovered during ‘open-ended lifestyle’ discussions with patients rather than through direct questioning.

What I noticed also is it is a habit among youngsters to use shisha. So, when I meet people of a certain age, I just ask them “Do you use shisha, do you go for shisha session?” [sic] (FG2, Maternity TDA nurse)

#### Providing support

Non-specialist healthcare professionals often described delivering initial brief interventions and education on the harms of non-cigarette tobacco. Two participants specifically highlighted educating chewing tobacco users about oral health and recognising symptoms of oral cancers. Non-specialist participants also reported ‘using motivational interviewing’ to ‘create and build motivation’ and address and identify stressors and triggers. Non-specialist healthcare professionals would also refer clients to stop smoking services, noting that they could provide greater specialist support:

[Smoking cessation services] are better equipped to offer tailored, culturally sensitive intervention and ongoing support, so it is a mix of both. We provide frontline support and education, and when more specialist care is needed we can refer (FG2, Pharmacist)

Most participants—both specialist and non-specialist—discussed the use of NRT with people using non-cigarette tobacco, noting its value in managing cravings. One specialist described using low-dose patches successfully with a client who occasionally smoked cigars. Vapes were also offered to clients who used cigars/cigarillos and shisha, as these can replicate the inhalation aspect of these products. However, participants expressed reluctance to provide vapes to people who used chewing tobacco, as they were not used to inhaling tobacco. Rather, nicotine gum and lozenges were viewed as more appropriate alternatives to mimic the chewing action. Nonetheless, it was acknowledged that nicotine gum cannot replicate the distinctive flavours of products such as paan.

I think the difference is that he [client who used chewing tobacco] didn’t get a vape which is something that would be given to somebody who was a smoker, a tobacco smoker. I was quite reluctant in giving him that because he doesn’t inhale so I didn’t want him to do that (FG3, TDA)

So, what works for [chewing tobacco users] is chewing gum, lozenges, or patch… we try to replace it with a similar habit(FG4, TDA)

One participant working with pregnant people noted that they used the national incentive scheme, an NHS scheme providing grocery vouchers to pregnant women if carbon monoxide levels remained under 4,^29^ with clients who used shisha. However, this programme could not be used among people who use chewing tobacco as these products do not expose people to CO.

### Barriers to providing non-cigarette tobacco cessation support

#### Client awareness of product constituents and health effects

Participants felt that many clients appeared to be unaware that the products that they used contained tobacco and, as a result, did not identify as tobacco users. Moreover, clients often were ‘not looking for support to quit because they don’t view it as a problem’ or did not realise that non-cigarette tobacco is harmful. Several participants noted that shisha and chewing tobacco, in particular, were commonly perceived by clients as safer alternatives to cigarettes.

Participants acknowledged that within certain communities some products, such as shisha and chewing tobacco, play a role in socialising situated within cultural identity, which contributes to perceptions that these products are not harmful.

It’s the social acceptance of chewing tobacco within these communities that has become one of the major hurdles that we have seen (FG4, TDA)

if it’s more from their cultures, then it’s believed to be not harmful because… they come from trees, so they’re not processed, they don’t have toxic chemicals…… But it’s that silent, slow killer (I2, TDA)

#### Client awareness of services

Some specialists perceived that, although their services support cessation for all forms of tobacco use, many people seemed unaware that help is available for non-cigarette products. Participants suggested that this may be partly due to service names, as they are often branded as ‘Stop Smoking Services’, which can give the impression that support is limited to cigarettes.

Two participants who work in areas with large South Asian communities commented that they rarely meet people who use chewing tobacco, noting that this may be due to a lack of outreach and awareness of support for these products among the local community.

Statistically you would be expecting a number, a significant proportion of people who may be utilising these products but I don’t think they’re necessarily filtering into our services (FG3, TDA)

#### Identifying use

Both specialist and non-specialist healthcare professionals reported that clients rarely ‘volunteer’ non-cigarette tobacco use unless specifically asked or it is ‘relevant to the topic that they are presenting with’.

[when discussing an interaction with a client] I asked her, ‘Do you engage in smoking?’ She said, ‘no’. ‘Do you use cigarettes?’ She said, ‘no’. I said, ‘Okay, in your party time, what do you like taking?’ She said, ‘I just use shisha’ (FG1, Nutritionist)

However, the way questions about tobacco use were phrased was seen as a key barrier. Standard screening tools typically ask only ‘Do you smoke?’. As many patients who use non-cigarette tobacco would not consider their product use smoking, they are unlikely to say yes to this question.

We probably will pick up any smoked tobacco if the question is asked correctly. So obviously we’re not going to be able to allow for the fact when you say do you smoke if somebody doesn’t see what they’re doing is smoking, they may well just say no (FG3, TDA)

Specialist participants also noted that screening for referrals is often undertaken by primary care practitioners such as GPs and nurses, many of whom are unfamiliar with non-cigarette tobacco products and therefore unlikely to ask about them explicitly.

The initial question is asked by admitting staff who are much more likely to be simplistic in their questioning (FG3, TDA)

Another challenge identified was terminology. Participants said they often lacked the right language to ask about certain products, particularly the many types of chewing tobacco, which are rarely referred to as ‘chewing tobacco’ by users, instead often clients will say *“*I’m chewing paan, the leaf, or betel nut*”*.

Paan can mean, if it’s translated, either ‘stuffed, packed’ or just that. So it’s a bit like saying, ‘Do you wear clothes?’ Yeah, but I might have just my underwear on. That’s clothes……We need to say, ‘What do you put in it?’ (I1, GP)

Some participants mentioned that they thought people were less likely to volunteer information on non-cigarette tobacco use because they are ashamed to seek help or talk about their dependence.

There’s a stigma, there’s a taboo, and it’s sociable, and it sort of doesn’t quite make sense… is it a stigma to people like me? A health professional? Or is it like, outside in the community? But it’s almost like, unless you’re in part of their gang, they wouldn’t go around- You know, the chewing gang. They’re not going to go and tell you (I1, GP)

#### Providing support

Specialist healthcare professionals noted that service commissioning can limit support for non-cigarette tobacco users, because some services are only funded to support smoked tobacco. Consequently, specialists in these services could only offer brief advice and suggest that clients buy NRT gum independently.

If it is not cigarette smoking, if it’s not combusted, then we cannot support them (I2, TDA)

None of the services [that the participant had worked for] helped with providing NRT because they’re not commissioned to do it (I2, TDA)

Most participants highlighted that resources and training also focus primarily on cigarette smoking, with very few materials tailored to non-cigarette tobacco. Consequently, professionals often adapt guidance for cigarettes (eg, the NCSCT treatment programme) or rely on their own judgement when developing treatment plans for people using non-cigarette tobacco. One non-specialist reported that they “don’t feel confident discussing these forms of tobacco” because “they are not covered in detail during standard smoking cessation training”. Another non-specialist stated that many of their colleagues were hesitant to prescribe NRT because “there’s less education around it” so “they’re not sure if they can use NRT” to support people using shisha and chewing tobacco.

Language barriers were another challenge, particularly for older people from certain communities who do not always speak English fluently. Some services use telephone translators; however, this can take time to book and therefore the window to engage clients is missed.

I’m unable to speak the language… we’ve got a translation person where they could ring up and then ask them to translate then and there. But then again, sometimes it can be a little bit nerve-wracking for them to go through these processes… when you think from their point of view, it shows that to get from A to B is going in a very, very different road, and you just give up halfway through (I2, TDA)

### Facilitators to providing non-cigarette tobacco cessation support

#### Engaging clients

Specialist healthcare professionals used a range of outreach methods, such as leafleting and running stalls at community events, “so that people are aware and they can come and talk”. Services also delivered targeted outreach, for instance, one specialist raised awareness about the effects of chewing tobacco among Bengali communities by setting up information stalls at local mosques. Specialists also highlighted the importance of engaging dentists, who may encounter teeth staining and mouth ulcers from chewing tobacco use, and encouraging them to refer patients to cessation services.

I personally tried my best to indulge into all the dentists across [local area], especially with those non-tradition tobacco leaflets (FG4, TDA)

The use of telephone and online appointments was reported by one specialist to have improved engagement, as they meant that clients who may struggle to come to face-to-face appointments have the flexibility to engage from home. This was caveated by another professional, however, who reported that face-to-face consultations are preferable for providing effective support. Postal NRT prescriptions were also said to improve the accessibility of NRT to some clients.

Having flexibility allows the client to feel more inclined to engage……. before, it was like, ‘if you’re not coming in, you’re not getting anything’ (I3, TDA)

Although some practitioners had issues with using telephone translators, others reported that they were ‘really useful’ and ‘very engaging’. Some services also had staff members who spoke multiple languages so could translate. Additionally, some services had translated leaflets and resources to improve wider engagement.

### Improving service provision for non-cigarette tobacco cessation support

An overview of key barriers and recommendations to improve the provision of non-cigarette tobacco cessation support is outlined in table 2.

**Table 2:** Overview of key barriers and recommendations to improve the provision of non-cigarette tobacco cessation support

Barrier	Recommendations*
**Awareness of services**: participants thought that many people who use non-cigarette tobacco are not aware that services may be able to support them in a quit attempt.	Conduct outreach among the local community, specifically advertising non-cigarette services Use community champions and existing networks to engage communities who use non-cigarette tobacco, for example, older South Asian communities. Reconsider service and staff titles to refer to tobacco and not smoking.
**Language barriers:** people who use non-cigarette tobacco struggle to engage with services if they do not speak English fluently.	Services should provide translated resources, for example, leaflets and informational videos. Services should have access to telephone translators.
**Screening tool:** current screening tools often only refer to smoking and not other types of tobacco.	Flexible screening tools should be developed to capture all types of tobacco use including smoked and smokeless.
**Commissioning**: not all services are commissioned to provide support for people using smokeless tobacco.	Local commissioners should consider expanding the criteria for support based on their local community’s needs.
**Clinical training**: there are few training sources available for healthcare professionals that cover non-cigarette tobacco use.	Training resources should be developed to educate healthcare professionals on non-cigarette tobacco. Training should also be available to all healthcare professionals, such as GPs and nurses, to increase primary healthcare practitioners’ awareness of non-cigarette tobacco.
**Clinical guidelines:** there is no official guidance on how to support people who use non-cigarette tobacco to quit.	Official clinical guidance on how to support cessation should be developed for cigars/cigarillos, shisha and chewing tobacco separately.

* The authors developed recommendations based on participants’ reported barriers and participants’ ideas for improving cessation support.

GPs, general practitioners.

#### Improving engagement

Specialists emphasised the need for greater public education to increase awareness of non-cigarette tobacco and its health risks. As above, developing informational leaflets in multiple languages was suggested to improve service outreach and community knowledge. One participant suggested that community champions could be used to improve engagement for certain communities, such as linking in with pre-existing South Asian women’s centres and groups.

Maybe getting a champion in that specific region…… I know that the Southeast Asian ladies [in local area] have once a week meetings……Reaching out to these communities to increase knowledge in these areas would be helpful because they speak the language, and they feel more comfortable reaching out to them (I2, TDA)

Participants stated that referral and screening processes need to be adapted to better capture non-cigarette tobacco use. Existing forms often fail to identify these products, limiting access to support.

Participants believed that all services should provide support for all tobacco products, not just cigarettes. They also suggested that service and practitioner titles should reflect this broader remit to make it clear that they offer non-cigarette tobacco support.

And also getting services to be commissioned for having a quit for smokeless tobacco so then- At the end of the day, It’s about quitting tobacco, which is the harmful product… that should be the main focus (I2, TDA)

#### Improving support

A key improvement mentioned by almost all participants was the need ‘to extend and diversify’ non-cigarette tobacco product-specific guidance and resources for practitioners, in order to ‘make support feel more inclusive and relevant’.

Training was considered important for all healthcare professionals, not just those specialising in tobacco cessation. GPs were highlighted as particularly important, given their frequent contact with patients. Follow-ups with people admitted to hospital were also mentioned as important.

It’s difficult because if someone is admitted to hospital, maybe it’s for one week, so it’s not for a long stay……. and there is no follow-up because when they are discharged, no one can talk about it anymore. The GP doesn’t talk about it, and nothing is done (FG2, Maternity TDA Nurse)

#### Acknowledging culture and identity

Many practitioners stressed that support needs to be sensitive, acknowledging the social and cultural significance of certain non-cigarette tobacco products. Support should consider what quitting means to the individual, including the potential sense of loss or grief.

Definitely you cannot take away that part of the culture without knowing and giving that some significance as to what they’re losing or grieving if they do (I1, GP)

## Discussion

Healthcare professionals have a range of experience supporting people to stop using non-cigarette tobacco products. Encountering non-cigarette tobacco use varied, but it was not uncommon for participants to encounter people using shisha, and chewing tobacco was frequently seen among services that were based in areas with South Asian communities who used outreach strategies. Cigars and cigarillos were infrequently mentioned by participants. Key barriers in providing cessation support included identifying use, services not being commissioned to support non-cigarette tobacco, and a lack of non-cigarette-specific training and guidance. To improve this, it was suggested that screening in healthcare settings should ask people about specific types of tobacco rather than just whether they smoke, and that specific guidance and treatment protocols should be developed for healthcare professionals to consult when they meet people who use non-cigarette tobacco. Additionally, it was thought that there should be improved awareness of the harms of non-cigarette tobacco and available cessation services among patients and those working in wider healthcare settings.

Cigars and cigarillos are the most common type of non-cigarette tobacco product used in England; however, few participants noted that they met people who used them, and those who did said that it was infrequent. This may be because people are rarely using cigars and cigarillos in isolation, as they often also smoke cigarettes.^3^ Some people may also conflate cigarettes and cigars/cigarillos, as these products all involve inhaling tobacco. Therefore, it is possible that people who use cigars or cigarillos only mention smoking or smoking cigarettes when talking to healthcare professionals. Additionally, certain services ran active outreach campaigns to engage with people who use chewing tobacco, but no services mentioned running specific outreach campaigns for cigars or cigarillos; therefore, they may not be capturing people who use these products exclusively.

Lack of awareness or acceptance of the harms of non-cigarette tobacco products emerged as a barrier that healthcare professionals perceived when attempting to engage people with cessation services. Similar to findings from previous research,^21–24^ people were often unaware of the potential harms of shisha and chewing tobacco, and therefore did not consider the need to stop using them. Information campaigns were suggested by participants as a way to improve awareness. Mass media campaigns highlighting the harms of smoking have been shown to improve knowledge about harms,^30^ although there is less evidence for non-cigarette tobacco campaigns. Campaigns for shisha and chewing tobacco in particular would need to be culturally appropriate, acknowledging the importance of certain products to communities and potentially using multilingual approaches so that they are better positioned to engage diverse communities. While language barriers can be an issue for all tobacco cessation support, it is especially present for non-cigarette tobacco use, which is disproportionately used among communities that do not have English as a first language.^31^

Identifying use was a key barrier, as current screening questions used by primary healthcare services typically only ask patients if they smoke, and many people who use shisha and chewing tobacco do not identify as ‘smokers’ or even as tobacco users. Screening should be expanded to ask participants about tobacco use more broadly, and consider using specific terms around shisha and chewing tobacco, as people do not always identify these as tobacco products. Co-design and community input could also be used to improve the terminology used to capture specific types of chewing tobacco products and provide a resource on the different names of these products. This could be a particularly helpful resource for healthcare professionals who rarely encounter non-cigarette tobacco and are therefore less aware of different terminologies used for these products.

When talking about providing support, specialists were often more confident in supporting people who used cigars/cigarillos than other types of non-cigarette tobacco, as they viewed them as more similar to cigarettes. The development of guidance on specific products such as shisha and chewing tobacco was thought to be key in improving cessation support provision. Although there are few national resources, some services had developed their own techniques to support people who want to quit these products. Collaboration between the NCSCT and these services could be used to create cessation support resources for healthcare professionals to consult nationwide. Some practitioners expressed uncertainty about the appropriateness of using vapes and NRT for non-cigarette tobacco, whereas others were happy to use these support strategies. A recent Cochrane review reported that cessation counselling, brief advice, varenicline and NRT all can help improve smokeless tobacco quit success compared with no support.^19^ Additionally, our participants reported good outcomes for vapes among people who use shisha, and for forms of NRT that replicate chewing and sucking (eg, gum, lozenges) among people using chewing tobacco, although further research is needed here. These findings could be integrated into new guidance to improve practitioners’ confidence in using different strategies to support non-cigarette tobacco cessation. As noted by participants, products such as chewing tobacco can hold a social and cultural value to certain communities; therefore, guidance development should integrate co-design to ensure that it acknowledges cultural sensitivities and does not increase shame or stigma.

Participants described being constrained by commissioning structures that only fund services for smoked tobacco. Services are commissioned by local councils and vary by area. As certain products, such as shisha and chewing tobacco, are known to be more prevalent among certain ethnic communities,^4^ commissioners should determine the need for broadening service criteria based on the diversity of communities in the local area, which could improve health equity in these areas.

### Strengths and limitations

A key strength of this study is the inclusion of both specialist and non-specialist healthcare professionals from diverse settings, offering a comprehensive view of current practice and barriers. However, most participants were based in London or other larger urban areas, which often have more diverse populations, and certain non-cigarette tobacco products (eg, chewing tobacco, shisha) tend to be more commonly used within particular cultural and ethnic groups. Services in these areas may therefore have greater exposure to non-cigarette tobacco use than those in less diverse or rural settings, which may limit the transferability of findings. Moreover, participants usually stated that they wanted to take part in this study due to a particular interest in non-cigarette tobacco; therefore, findings may represent the experiences of a particularly motivated group of healthcare professionals. Findings also represent professional perspectives only, and may not capture the experiences or priorities of non-cigarette tobacco users themselves. Further qualitative work exploring users’ perspectives is needed to understand motivations, perceived risks and barriers to accessing cessation support.

## Conclusions

There is substantial variation in the availability of, and healthcare professional confidence in providing, support for non-cigarette tobacco cessation across England. Amending screening tools, developing clinical training and guidance, and expanding access to services for all types of tobacco use could improve support provision and equity in supporting people to stop tobacco use.

## Data Availability

No data are available.
